# Detecting local risk factors for residual malaria in northern Ghana using Bayesian model averaging

**DOI:** 10.1186/s12936-018-2491-2

**Published:** 2018-09-29

**Authors:** Justin Millar, Paul Psychas, Benjamin Abuaku, Collins Ahorlu, Punam Amratia, Kwadwo Koram, Samuel Oppong, Denis Valle

**Affiliations:** 1 Emerging Pathogens Institute, University of Florida, Gainesville, USA; 2 Noguchi Memorial Institute for Medical Research, College of Health Sciences, University of Ghana, Legon, Ghana; 3 National Malaria Control Programme, Public Health Division, Ghana Health Service, Accra, Ghana

**Keywords:** Risk factors, Bayesian model averaging, Nonlinear patterns, Statistical methods

## Abstract

**Background:**

There is a need for comprehensive evaluations of the underlying local factors that contribute to residual malaria in sub-Saharan Africa. However, it is difficult to compare the wide array of demographic, socio-economic, and environmental variables associated with malaria transmission using standard statistical approaches while accounting for seasonal differences and nonlinear relationships. This article uses a Bayesian model averaging (BMA) approach for identifying and comparing potential risk and protective factors associated with residual malaria.

**Results:**

The relative influence of a comprehensive set of demographic, socio-economic, environmental, and malaria intervention variables on malaria prevalence were modelled using BMA for variable selection. Data were collected in Bunkpurugu-Yunyoo, a rural district in northeast Ghana that experiences holoendemic seasonal malaria transmission, over six biannual surveys from 2010 to 2013. A total of 10,022 children between the ages 6 to 59 months were used in the analysis. Multiple models were developed to identify important risk and protective factors, accounting for seasonal patterns and nonlinear relationships. These models revealed pronounced nonlinear associations between malaria risk and distance from the nearest urban centre and health facility. Furthermore, the association between malaria risk and age and some ethnic groups was significantly different in the rainy and dry seasons. BMA outperformed other commonly used regression approaches in out-of-sample predictive ability using a season-to-season validation approach.

**Conclusions:**

This modelling framework offers an alternative approach to disease risk factor analysis that generates interpretable models, can reveal complex, nonlinear relationships, incorporates uncertainty in model selection, and produces accurate predictions. Certain modelling applications, such as designing targeted local interventions, require more sophisticated statistical methods which are capable of handling a wide range of relevant data while maintaining interpretability and predictive performance, and directly characterize uncertainty. To this end, BMA represents a valuable tool for constructing more informative models for understanding risk factors for malaria, as well as other vector-borne and environmentally mediated diseases.

**Electronic supplementary material:**

The online version of this article (10.1186/s12936-018-2491-2) contains supplementary material, which is available to authorized users.

## Background

In spite of significant global reductions in malaria transmission and prevalence over the past decade [1], many districts and municipalities across sub-Saharan Africa continue to experience high malaria burden [2, 3]. In several instances, residual malaria transmission has persisted despite widespread coverage of conventional malaria interventions, such as insecticide-treated bed netting (ITN) and indoor-residual spraying of insecticides (IRS) [4, 5]. An important factor contributing to residual malaria transmission is a high degree of spatial heterogeneity [5]. Malaria prevalence can differ dramatically [6], even over relatively short distances [7], which has the potential to undermine universal intervention guidelines [8]. Similarly, some subpopulations might have a substantially higher malaria risk than other groups. Identifying these hotspots and hot-pops is critical for developing targeted approaches to reduce malaria burden and guide holoendemic areas towards malaria elimination [7, 9].

Local risk factors for malaria can be difficult to characterize due to the wide range of variables that can be relevant to malaria epidemiology [10]. Studies on malaria risk factors have often focused on particular types or categories of variables, such as models based on environmental data [11–13], or demographic and socio-economic factors [14–17]. However, as information becomes more accessible and available at finer geographic and temporal resolutions, malaria risk models have sought to incorporate a greater variety of explanatory data [18–21]. Additionally, the importance of complex patterns, such as nonlinear relationships and seasonally-dependent shifts, has emerged as a significant component to modelling malaria risk [22, 23].

Incorporating a wider range of explanatory information into disease risk factors models can be difficult when using traditional statistical approaches such as standard logistic regression. Having a large number of predictors or independent variables (i.e. potential risk factors) can lead to overfitting [24], which can decrease the accuracy of out-of-sample predictions and increase the probability of detecting spurious relationships, which in the context of disease risk factor analysis can undermine the applicability towards guiding interventions. Additionally, traditional statistical models often make critical assumptions, such as linearity. These shortcomings have led to the application of sophisticated variable selection methods, which are able to incorporate more independent variables and model complex relationships without sacrificing forecasting accuracy by reducing dimensionality. Examples of variable selection methods that have been used for malaria-related data include stepwise regression [20], ridge regression [25], and Lasso regression [26, 27].

One limitation of these approaches for variable selection is they do not account for uncertainty in the selection process, which can produce overconfident predictions. Consider the following contextualized example provided by Hoetling et al. [28]: a researcher has gathered a comprehensive data set on potential risk factors of malaria, and wants to construct a model in order to compare risk factors and make predictions. They use a variable selection procedure, which identifies a specific model, M^*^, as having the best fit based on some information criterion, which is then used to compare risk factors, make predictions, and inform interventions. Suppose that there exists an alternative model, M^**^, which has nearly as good of fit but consists of a different set of covariates and produces different effect sizes and/or predictions. In this case, the researcher should have less certainty in M^*^. Hoeting et al. [28] demonstrates that this scenario where uncertainty in model selection is ignored is very common and unfortunately typical variable selection methods do not provide a mechanism for incorporating this uncertainty.

Bayesian model averaging (BMA) is an alternative approach to variable selection which fully accounts for uncertainty associated with the model selection process [29]. Previous studies outside of the field of disease control have demonstrated that BMA often outperforms other methods of variable selection [30–32]. This technique has been adopted in many modelling applications [33], such as weather forecasting [34, 35], phylogenetics [36], and hyperspectral image analysis [32, 37]. While BMA is not new to modelling disease risk factors [38, 39], recent applications (i.e. in the past 15 years) are uncommon, and to our knowledge BMA has yet to be used in the context of malaria or other arthropod-transmitted diseases. Given the wide variety of factors that contribute to malaria, the increased attention to complex patterns, and the increasing availability of data, BMA could represent a valuable statistical tool for enhancing risk factor models and designing targeted interventions.

In this study, BMA was used to identify the underlying factors that shape the spatiotemporal patterns of malaria prevalence in a district located in the Guinea savannah zone of northern Ghana that experiences high seasonal malaria transmission [40, 41]. The article demonstrates how BMA can be used to identify seasonal differences and nonlinear relationships in malaria risk factors, compare the performance of BMA to standard logistic and Lasso regression, and describe how BMA results can be useful for designing targeted malaria intervention strategies.

## Methods

### Site description

**Fig. 1:**
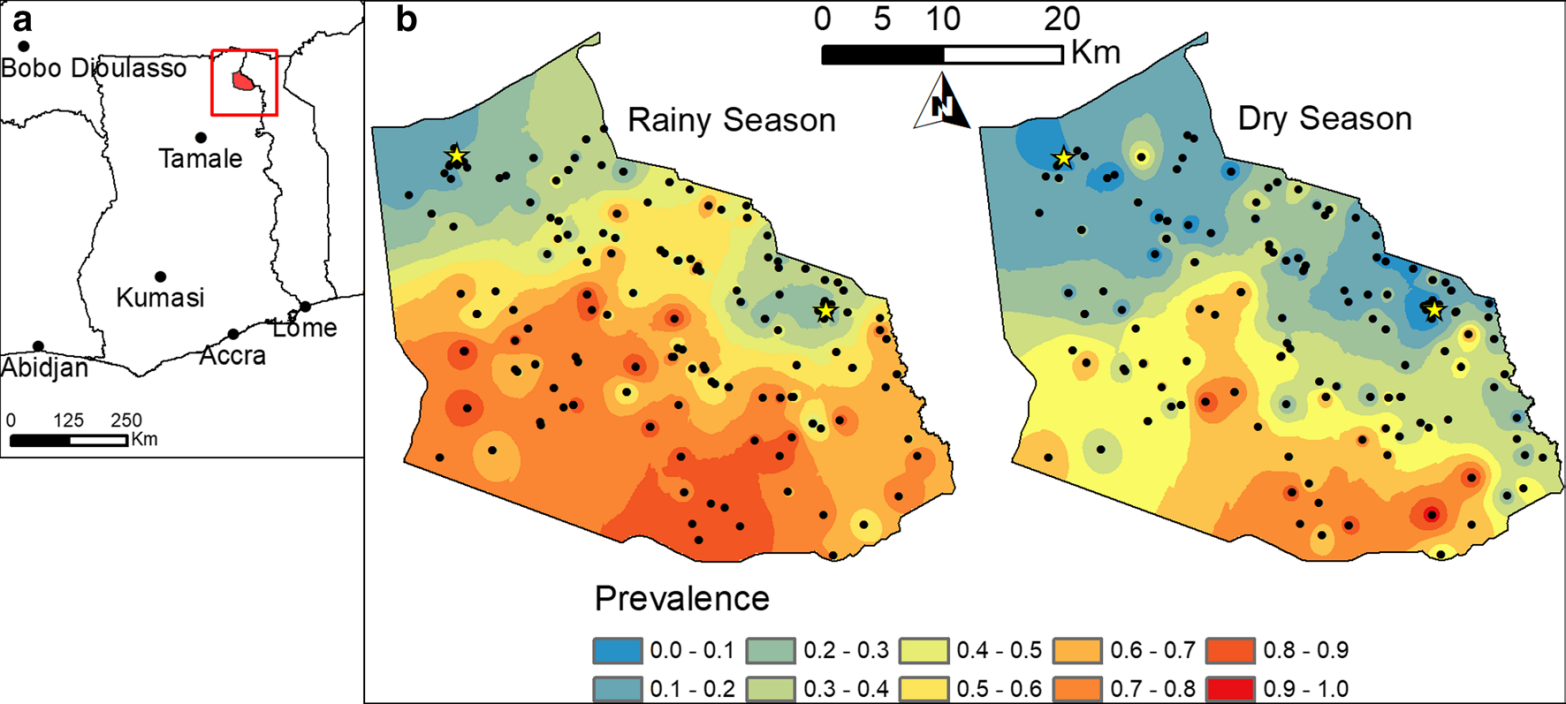
Map of study district, Bunkpurugu-Yunyoo, in northern Ghana (red polygon show in insert map). Interpolations depict malaria prevalence in young children (ages 6–59 months) in Bunkpurugu-Yunyoo, Ghana during the rainy and dry seasons in the left and right maps, respectively. Six biannual surveys were collected from 2010 to 2013 and pooled by season. Black circles denote the sampled communities and yellow stars denote local urban centers. Interpolations were made using inverse-distance weighted function in ArcGIS 10.3

Data were collected from the Bunkpurugu-Yunyoo district, Northern Region, which is in the Guinea savannah zone of northeastern Ghana and experiences recurring high levels of seasonal malaria transmission (Fig. 1). Two highly efficient malaria vectors predominate in this area, namely *Anopheles gambiae* sensu stricto (s.s.) and *Anopheles funestus* [42]. During the study period coverage of long-lasting insecticide-treated bed net (LLINs) was greater than 75%, having benefitted from two mass distribution campaigns in 2010 and 2012. Furthermore, annual IRS campaigns were conducted in 2011 and 2012 using alphacypermethrin 0.4% WP (ICON^®^10CS, Syngenta, Basel Switzerland), with a second application of IRS provided in the dry season in the eastern portion of the district.

The district is composed of rural communities supported by small-scale farming and herding, and two modest urban centers: Bunkpurugu (population: 7436) and Nakpanduri (population: 5783). The major ethno-linguistic groups are the Bimoba (approximately 60%) and Konkomba (approximately 30%) with smaller populations of Mamprusi, Kusasis, Dagombas, Fulanis and others. The Bimoba tend to predominate in the higher ground of the north and east portions of the district, including the two urban areas, while the Konkomba are more prevalent in the lower lying area of the south and west, where they graze their cattle in the riverine plains. The Konkomba, who tend to be a geographically and economically marginalized group across northern Ghana, are recognized as more culturally conservative and in general tend to be less educated [43].

### Data collection

The individual-level longitudinal dataset was collected in the course of operations research on IRS, which was conducted by the University of Ghana with the support of the President’s Malaria Initiative [44, 45]. The current study, which was carried out at the University of Florida in collaboration the University of Ghana, consisted fundamentally of enhancing that original dataset with remote-sensed variables and conducting follow-on analyses to address a different set of research objectives.

Children between the ages of 6 to 59 months were surveyed in six biannual surveys, three during the rainy season (late October to November) and three during the dry season (late March to April), from 2010 to 2013. A new representative sample was selected for each survey using a multi-stage randomized cluster sampling technique. Probability proportional to size estimates were used to randomly select representative communities based on a Ghana Health Service roster of communities in the district. This sample covered approximately 20% of the under-five population in each survey, based on 2010 census data. Individuals under 6 months old were removed from this analysis to eliminate the influence of maternal immunity. Each survey was conducted over a 3-week period. Malaria status was assessed via blood-film microscopy. The survey also captured data on relevant demographic, socioeconomic, and malaria intervention variables (Table 1), using a modified Malaria Indicator Survey questionnaire. GPS coordinates were recorded for a central point in each community center. The original dataset was enhanced by collecting additional information on environmental variables using GIS software (ArcMap 10.4) and freely available remote sensing sources (Table 2). Childhood malaria prevalence in this district exhibited a high degree of spatial heterogeneity over the study period, in both the rainy and dry seasons (Fig. 1).

**Table 1:** Potential risk or protective covariates collected from surveys

Variable	Details
Demographic and socio-economic	
Age	From 6 to 59 months old
Caretaker’s education	Binary variable; either (1) for high school education and above or (0) otherwise
Caretaker’s age	In years
Ethnicity	Four groups; (1) Bimoba, (2) Konkomba, (3) Mamprusi, and (4) Other, based on language of caretaker
Farming caretaker^a^	Binary variable; either caretaker occupation being farming (1) or otherwise (0)
Gender	Binary variable; either male (1) or female (0)
Surface water source	Binary variable; either (1) source of drinking water from exposed surface water or (0) otherwise
Thatch roofing	Binary variable; either housing structure had a thatched roof (1) or otherwise (0)
Wealth quintile	Constructed from multiple variables, using the methodology of the Ghana Demographic Health Survey (2008) [80]
Malaria intervention	
Health insurance—personal	Binary variable; either personal access to health insurance (1) or not (0)
Health insurance—community	Binary variable; either (1) for ≥ 80%^b^ community coverage of sampled population or (0) otherwise
IRS in past 7 months^a^	Binary variable; either individual household having been treated with IRS in past 7 months (1) or not (0)
IRS in past year	Binary variable; either individual household having been treated with IRS in past year (1) or not (0)
Indoor residual spraying (IRS)—community coverage	Binary variable; either (1) for ≥ 80%^b^ community coverage or (0) otherwise
Insecticide treated nets (ITN)—personal	Binary variable; either (1) if net was used in previous night or (0) otherwise
ITN—community coverage	Binary variable; either (1) for ≥ 80^b^ % community coverage or (0) otherwise
Personal medication use	Binary variable; either (1) used in the past 2 weeks or (0) otherwise

^a^Removed from models due to high correlations (R^2^ ≥ 0.49) with one or more other variables

^b^Based on targets from Roll Back Malaria

**Table 2:** Potential risk or protective covariates collected from remote sensing and GIS-based sources

Variable	Source/satellite	Details
Distance to health facility	GIS-derived	Euclidean distance from active health facility at time of survey (based on survey location)
Distance to main roads	GIS-derived [81]	Euclidean distance from major roads
Distance to urban centers	GIS-derived	Euclidean distance from center with population ≥ 5000 individuals
Distance to water bodies	GIS-derived [82]	Euclidean distance from rivers and standing water bodies
Elevation	CGIAR SRTM [83]	Meters above sea level
Land surface temperature—day^a^	NASA (Terra) MOD13A3 (Aqua) MYD13A3 [84]	Average monthly daytime temperature (in degrees Celsius) 30 days prior to a survey
Land surface temperature—night	NASA (Terra) MOD13A3 and (Aqua) MYD13A3 [84]	Average monthly nighttime temperature (in degrees Celsius) 30 days prior to a survey
Normalized difference vegetative index^a^	NASA (Terra) MOD13A3 and (Aqua) MYD13A3 [85]	The maximum monthly index 30 days prior to a survey
Population density	WorldPop [86]	Population density per 100 m grid, log-transformed
Population density (≤ 5 y.o.)^a^	WorldPop [86]	Population under 5 years of age density per 100 m grid, log-transformed
Rainfall (historical)^a^	WorldClim [87]	Average of the cumulative sum of precipitation from 3 to 1 month prior to the survey date from past 50 years
Rainfall (current)^a^	FEWSNET [88]	Average of the cumulative sum of precipitation from 3 to 1 month prior to survey
Slope	GIS-derived (from elevation)	

^a^Removed from models due to high correlations (R^2^ ≥ 0.49) with one or more other variables

Correlations between all potential risk factors were calculated, and in cases of high correlations (R^2^ > 0.49), a single representative covariate was selected (see Additional file 1). Selection of these covariates was based on the relevance of each covariate to malaria epidemiology and intervention strategies. The covariates dropped from all models were farming caretakers, indoor residual spraying (IRS) in past 7 months, average daytime land surface temperature, normalized difference vegetation index (NDVI), cumulative rainfall, and historical precipitation trends. Because BMA requires each individual to have information for all covariates, all individuals with at least one covariate with missing data were dropped from the analysis. As a consequence, all analyses were based on 10,029 children (84.0% of total dataset). These data were distributed across 80 communities in the first survey and 71 communities in each of the subsequent surveys. The number of individuals in each survey ranged from 1341 to 1788.

### Statistical methods

#### Base model

All malaria risk models were constructed using the same general Bayesian framework. Let *y*_*ijt*_ be the binary microscopy outcome (1 = positive, 0 = negative) for individual *i* in community *j* at time *t*. This variable was modelled using a Bayesian probit regression model, assuming that:

$$\begin{array}{l} y_{ijt} = 1\quad {\text{if}}\,z_{ijt} > 0 \hfill \\ y_{ijt} = 0\quad {\text{otherwise}} \hfill \\ \end{array}$$

In other words, individual *i* in community *j* at time *t* is positive for malaria only if *z*_*ijt*_ is greater than zero. This is determined by:

$$z_{ijt} \sim N\left( {x_{ijt}^{T} \beta ,1} \right)$$

where *x*_*ijt*_^*T*^ is a vector of the intercept and potential risk factors, and *β* is a vector with the corresponding regression parameters. Finally, the priors were specified as:

$$\beta \sim N\left( {0,\sigma^{2} \varSigma } \right)$$

$$\sigma \sim Unif\left( {0,100} \right)$$

where the matrix Σ in the prior for *β* is a diagonal matrix with $$diag\left( \varSigma \right) = \left[ {\begin{array}{*{20}c} {100} & 1 & \ldots & 1 \\ \end{array} } \right]$$. Similar Bayesian regression frameworks have been used in disease risk factors analyses, including for HIV and tuberculosis [46, 47], as well as malaria [48].

#### Complex models: seasonal differences and nonlinear associations

In addition to the general risk factor regression, extended versions of the base model were created by including additional derived covariates in order to describe complex patterns. First, a model was constructed to evaluate whether the effect of risk factors differed between the dry and rainy seasons. For example, distance to the nearest health facility may be a strong risk factor in the rainy season but may be an irrelevant covariate during the dry season. This was modelled by including additional elements in the design vector $x_{ijt}^{T}$ representing the interaction of each covariate with the binary variable representing the rainy season. This model allows the parameter estimates for each covariate to vary by season. Risk or protective factors that vary substantially with season may suggest that different malaria intervention strategies could be required for each season.

Finally, this framework was used to describe potential nonlinear patterns in two relevant continuous variables, distance to nearest urban centre and distance to nearest health facility, through the use of linear splines. These variables were selected based on outcomes from the base model and their applicability towards design interventions. The creation of linear splines consists of first a set of *m* values within the domain of the covariate *x*, referred to as knots $k_{1} , \ldots , k_{m}$. For which knot, a “new” derived covariate *x*_*d*_ is created in the following way:

$$x_{d} = \left\{ {\begin{array}{ll} 0, &\quad x < k_{d} \\ x - k_{d} ,&\quad x \ge k_{d} \\ \end{array} } \right.$$

resulting in *m* additional derived variables for each splined covariate. Knot values were selected at the 20, 40, 60 and 80% quantiles of the observed variables. Including these splines allows the effect of these variables to shift at the knot values, which can reveal nonlinear associations in the specified risk factors. Seasonal interaction terms were also included in this model, which allowed these nonlinear patterns to also differ in each season.

#### Bayesian model averaging

Each of the models discussed above can be fitted using a Markov chain Monte Carlo (MCMC) algorithm. Variable selection was incorporated into this MCMC algorithm by implementing a reversible jump MCMC [49]. The MCMC is initialized with a model containing a subset of the possible covariates. At each iteration of the MCMC a new candidate model is proposed using a randomly selected move; either a birth (addition of a new covariate), death (removal of an included covariate), or swap (switching an included covariate with an excluded covariate). The candidate model is then either accepted or rejected based on the marginal log-likelihood. Informative covariates (and combination of covariates) will have a tendency to increase the marginal likelihood, and therefore tend to be retained in the selection process, while less informative covariates are more likely to be excluded.

The marginal probability associated with a particular model *M*_*q*_, defined by the subset of covariates q, can be calculated in closed form after integrating out the associated regression parameters $\varvec{\beta}_{\varvec{q}}$. This is given by:

$$p\left( {M_{q} |\varvec{z},\sigma^{2} } \right) \propto \int N\left( {\varvec{z}|\varvec{X}_{q}\varvec{\beta}_{\varvec{q}} ,\varvec{I}} \right){\text{N}}\left( {{\varvec{\upbeta}}_{\varvec{q}} |0,\sigma^{2} {\varvec{\Sigma}}} \right)d\varvec{\beta}_{\varvec{q}}$$

$$\propto \left( {\sigma^{2} } \right)^{{ - \frac{{p_{q} + 1}}{2}}} exp\left( { - \frac{1}{2}\left[ { -\varvec{\mu}_{\varvec{q}}^{\varvec{T}} {\mathbf{T}}_{\varvec{q}}^{ - 1}\varvec{\mu}_{\varvec{q}} } \right]} \right)\left| {\varvec{T}_{\varvec{q}} } \right|^{{\frac{1}{2}}}$$

where *p*_*q*_ is the number of covariates in subset q, ${\mathbf{T}}_{\varvec{q}}^{ - 1} = \left\{ {\varvec{X}_{\varvec{q}}^{\varvec{T}} \varvec{X}_{\varvec{q}} + \frac{1}{{\sigma^{2} }}{\varvec{\Sigma}}_{\varvec{q}}^{ - 1} } \right\}$ and $\varvec{\mu}_{\varvec{q}} = {\mathbf{T}}_{\varvec{q}} \varvec{X}_{\varvec{q}}^{\varvec{T}} \varvec{z}$.

The prior for each model were then set to $$p\left( {M_{q} } \right) \propto \left( {\begin{array}{*{20}c} P \\ {p_{q} } \\ \end{array} } \right)^{ - 1} ( {P + 1} )^{ - 1}$$, where P is the overall number of covariates. In this expression, $$\left( {\begin{array}{*{20}c} P \\ {p_{q} } \\ \end{array} } \right)$$ counts all the possible combinations of *p*_*q*_ elements out of P and $\frac{1}{P + 1}$ is a discrete uniform distribution for all possible number of covariates 0, …, *P*.

As mentioned above, the algorithm explores model space by randomly proposing the birth of a new covariate or the death or swap of an existing covariate. These proposed moves are then accepted or rejected using a standard Metropolis–Hastings acceptance ratio given by:

$$\hbox{min} \left\{ {1,\frac{{p\left( {M_{{q^{ *} }} |\varvec{z},\sigma^{2} } \right)p\left( {M_{{q^{ *} }} } \right)}}{{p\left( {M_{q} |\varvec{z},\sigma^{2} } \right)p\left( {M_{q} } \right)}}} \right\} = \hbox{min} \left\{ {1,\frac{{e\left( {\sigma^{2} } \right)^{{ - \frac{{p_{{\varvec{q}^{ *} }} + 1}}{2}}} exp\left( { - \frac{1}{2}\left[ { -\varvec{\mu}_{{\varvec{q}^{ *} }}^{\varvec{T}} {\mathbf{T}}_{{\varvec{q}^{ *} }}^{ - 1}\varvec{\mu}_{{\varvec{q}^{ *} }} } \right]} \right)\left| {\varvec{T}_{{\varvec{q}^{ *} }} } \right|^{{\frac{1}{2}}} }}{{\left( {\sigma^{2} } \right)^{{ - \frac{{p_{q} + 1}}{2}}} exp\left( { - \frac{1}{2}\left[ { -\varvec{\mu}_{\varvec{q}}^{\varvec{T}} {\mathbf{T}}_{\varvec{q}}^{ - 1}\varvec{\mu}_{\varvec{q}} } \right]} \right)\left| {\varvec{T}_{\varvec{q}} } \right|^{{\frac{1}{2}}} }} \times R} \right\}$$

where *R* is typically equal to 1 and $M_{{q^{ *} }}$ and *M*_*q*_ are the proposed and current models, respectively.

This approach was used to fit a customized Gibbs sampler (see Additional file 1) using R software (v3.3.1) [50]. Each model was run for 10,000 iterations with the first 1000 iterations dropped to account for the burn-in period. Convergence on the parameter estimates was confirmed using trace plots. Similar to traditional Bayesian regression, the regression coefficient (*β*) for each covariate is estimated based on posterior draws and considered statistically significant if the 95% credible interval did not contain zero. Note that this is the “model averaging” component of BMA, as individual posterior samples are based on different parameter spaces. This allows for the uncertainty associated with variable selection to be incorporated into parameter estimation. More in-depth descriptions of this model and how it is fit are provided by Zhao et al. [32] and Denison [51].

### Out-of-sample predictions

As illustrated in the preceding sections, allowance for greater model flexibility can be achieved through the additional of several derived covariates and their associated parameters. Specifically, the base model contained 29 covariates, adding interaction terms increased the number of covariates to 56, and adding linear splines expanded the model to include a total of 73 covariates. Increasing the number of parameters in a model can lead to overfitting, making variable selection an increasingly important task. To assess the out-of-sample performance of BMA, we performed predictions by training the model on data from a particular year and estimating malaria status for a future year. Due to high seasonality in malaria risk in the district, only same-season predictions were considered (i.e. rainy season predictions were based on a rainy season training dataset). These predictions were compared to standard logistic regression, as well as least absolute shrinkage and selection operator (Lasso) regression. Lasso is an alternative method for variable selection which has been shown to improve out-of-sample predictions [52]. To demonstrate how these models performed relative to the number of covariates, season-to-season predictions for the base model and the extended model which contained seasonal interactions and spline terms were performed. Out-of-sample predictive skill was evaluated based on the sum of the log likelihood, where the model with the largest log likelihood sum was considered to have the best predictive ability.

## Results

### Descriptive analysis

There was a slight decreasing trend in malaria prevalence over the course study, however the distribution of seasonal community prevalence remained relatively consistent over the course of the study (see Additional file 1). Mean community prevalence (and interquartile ranges) in the three rainy season surveys were 0.57 (0.39–0.75), 0.52 (0.33–0.73), and 0.46 (0.27–0.61), whereas in the three dry season surveys these values were 0.35 (0.15–0.50), 0.31 (0.14–0.47), and 0.23 (0.10–0.33). The parasitaemia rate remained high during the final rainy season despite high coverage of ITNs and 2 years of IRS, highlighting the importance of devising complementary malaria control strategies based on the local risk factors.

### Risk factor outcomes

#### Base model

**Fig. 2:**
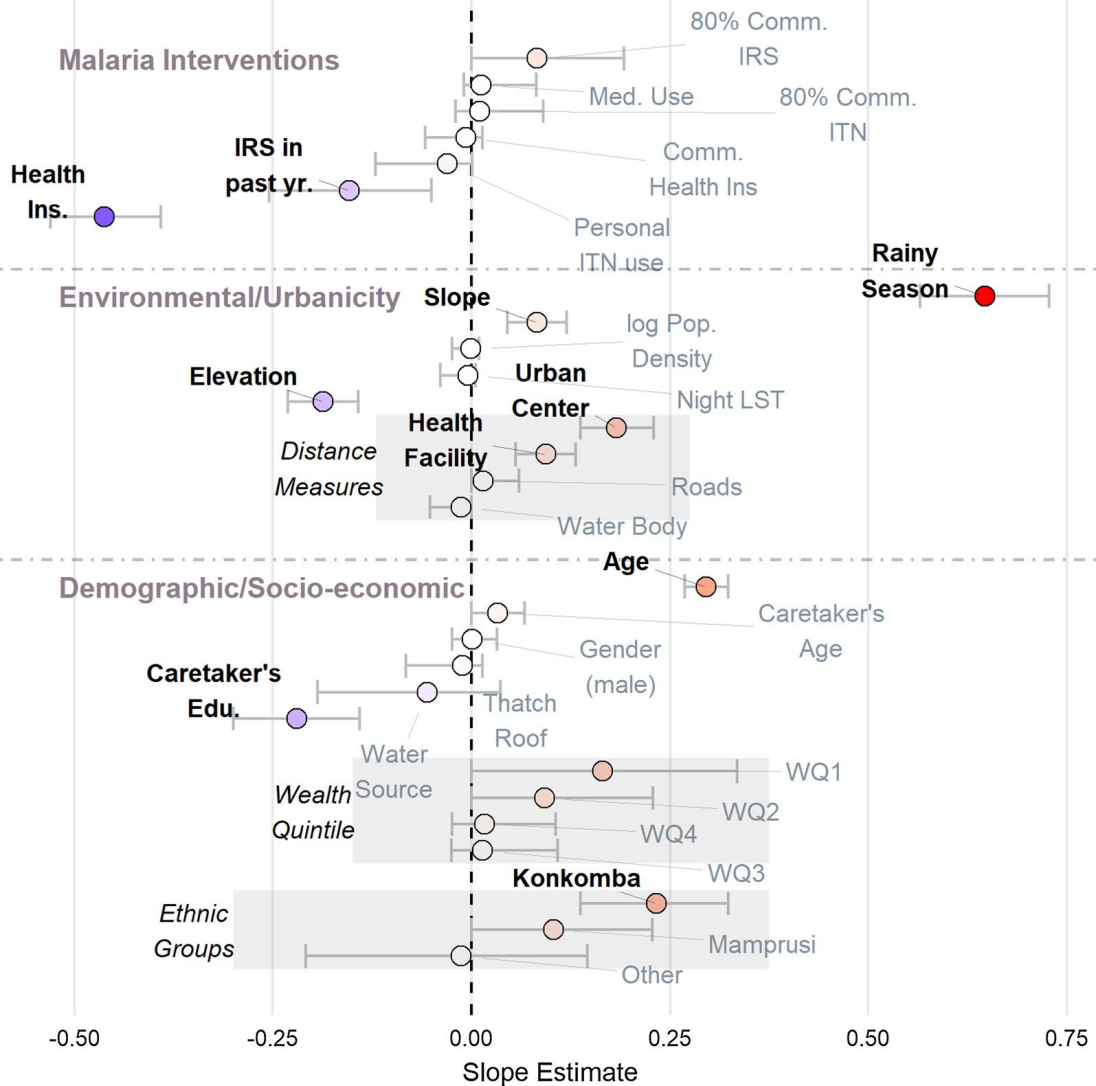
Mean slope estimates (circles) and 95% credible intervals (horizontal grey bars) from probit regression parameters. Variables whose credible intervals do not include zero are considered significant (labelled in bold). Risk factors (positive slopes) and protective factors (negative slopes) are shown in red and blue, respectively

The basic risk factor model with BMA variable selection detected that many expected, classic patterns of malaria risk factors are present amongst the early childhood populations in Bunkpurugu-Yunyoo (Fig. 2). The strongest risk factor associated with malaria infection was rainy season (mean regression coefficient equal to 0.647 with a credible interval (CI) of 0.565–0.728), as evident in the prevalence maps (Fig. 1). Age was also a significant risk factor (0.296, CI 0.268–0.324), as would be expected among young children (i.e. less than 5 years old) in an area of stable, holoendemic malaria. Among the distance measures, distance to nearest health facility (0.094, CI 0.056–0.131) and urban centers (0.183, CI 0.137–0.229) were significant risk factors, whereas distance to nearest road (0.014, CI 0.00–0.060) and water body (− 0.013, CI − 0.052 to 0.00) had little to no effect. The Konkomba communities experienced significantly higher malaria risk (0.233, CI 0.137–0.323), relative to the Bimoba, and generally had a high mean prevalence overall. Note that this represents the risk associated with ethnicity are adjusting for other covariates in the model, such as education, wealth, and elevation. Statistically significant protective factors were access to health insurance (− 0.463, CI − 0.530 to − 0.391) and mother’s education (− 0.220, CI − 0.300 to − 0.141). Elevation was a significant factor, however given the relatively narrow range in elevations (135–449 meters above sea level) this is likely a consequence of the two urban centers being in higher elevation, not because of high-altitude effects on local climate. IRS in the past year was also a significant protective factor (− 0.154, CI − 0.254 to − 0.050). The categorical variables for wealth quintiles did not have statistically significant effects individually, however as a group these variables indicated that the lower wealth quintile groups (below median and well below median) were positively associated with malaria prevalence.

#### Seasonal differences

**Fig. 3:**
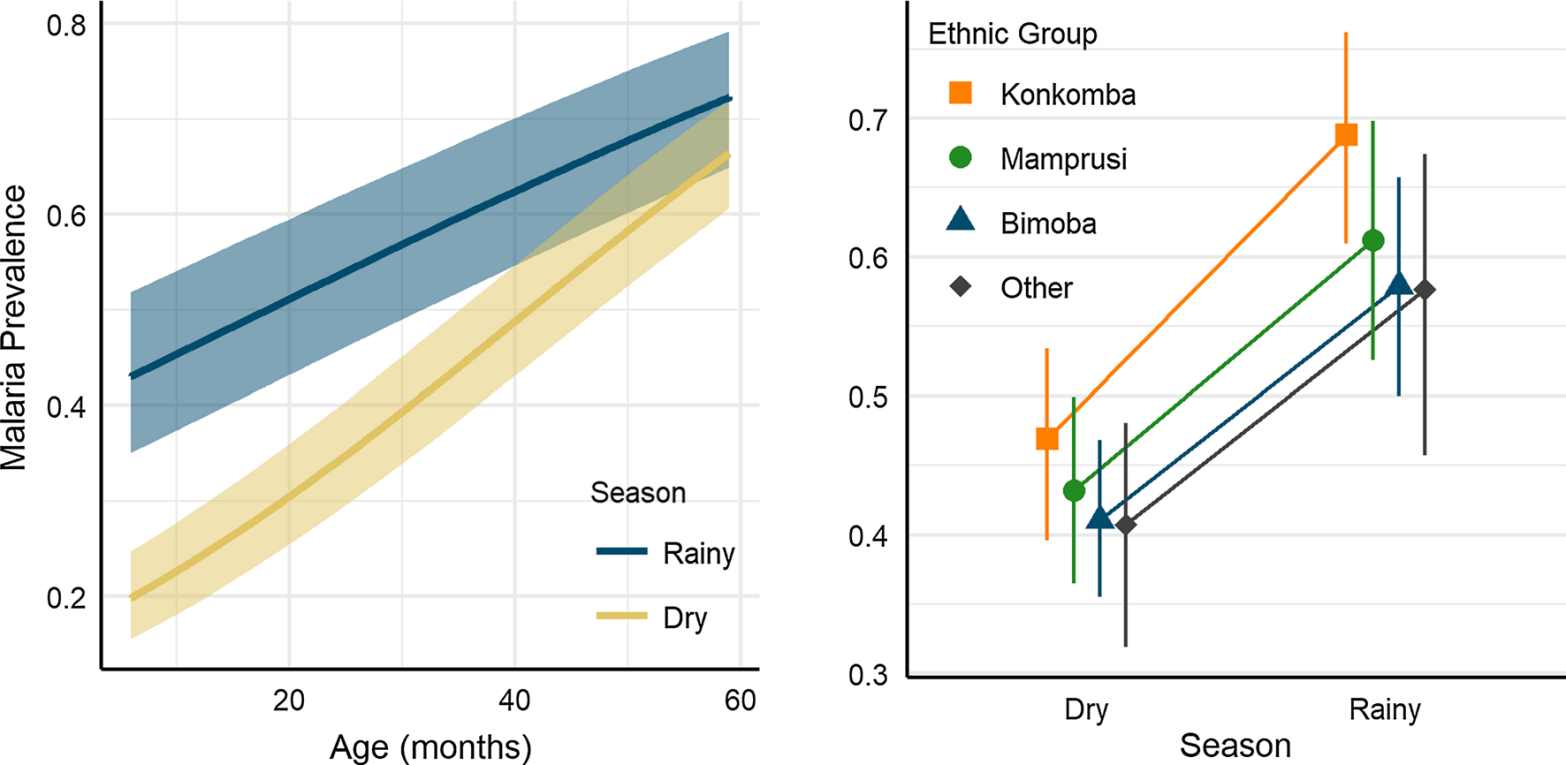
Modelled patterns in malaria risk factors based on Bayesian probit regression containing seasonal interaction terms. The left panel depicts mean slope estimate (lines) and 95% credible intervals (polygons) for the predicted malaria prevalence based on age in the rainy and dry seasons. The right panel depicts the mean (points) and 95% credible intervals (vertical bars) for the predicted malaria prevalence based on ethnic group in the rainy and dry seasons

Modelling malaria risk with seasonal interaction terms (see Additional file 1 for regression coefficients) suggested most risk factors did not exhibit prominent differences between the rainy and the dry seasons, with a few notable exceptions. Age was an important risk factor for malaria in both seasons, however the slope estimate for this parameter was significantly lower in the rainy season than in the dry season, as illustrated in Fig. 3. These patterns suggest that while all ages experience higher malaria burden in the rainy season, children in the upper end of the observed age range (50–59 months old) experienced nearly the same predicted prevalence in the dry season as they did in the rainy season (Fig. 3). Another important finding refers to ethnicity. All ethnic groups experienced increased malaria burden in the rainy season, however predicted mean prevalence based on seasonal-ethnicity interaction terms indicate that the increase in malaria prevalence during the rainy season was more intense for the Konkomba communities than for the other ethnic groups (Fig. 3). For example, the odds-ratio associated with the effect of Konkomba ethnicity compared to Bimoba ethnicity increased from 1.27 in the dry season to 1.60 in the rainy season. By comparison, the odds-ratio associated with the effect of Mamprusi ethnicity compared to Bimoba ethnicity were 1.09 and 1.15 in the dry and rainy seasons, respectively. Other marginal differences included health insurance, which was less significant of a protective factor in the rainy season, and personal medication use, which was a moderate risk factor in the dry season but had relatively no influence in the rainy season (see Additional file 1).

#### Nonlinear associations

**Fig. 4:**
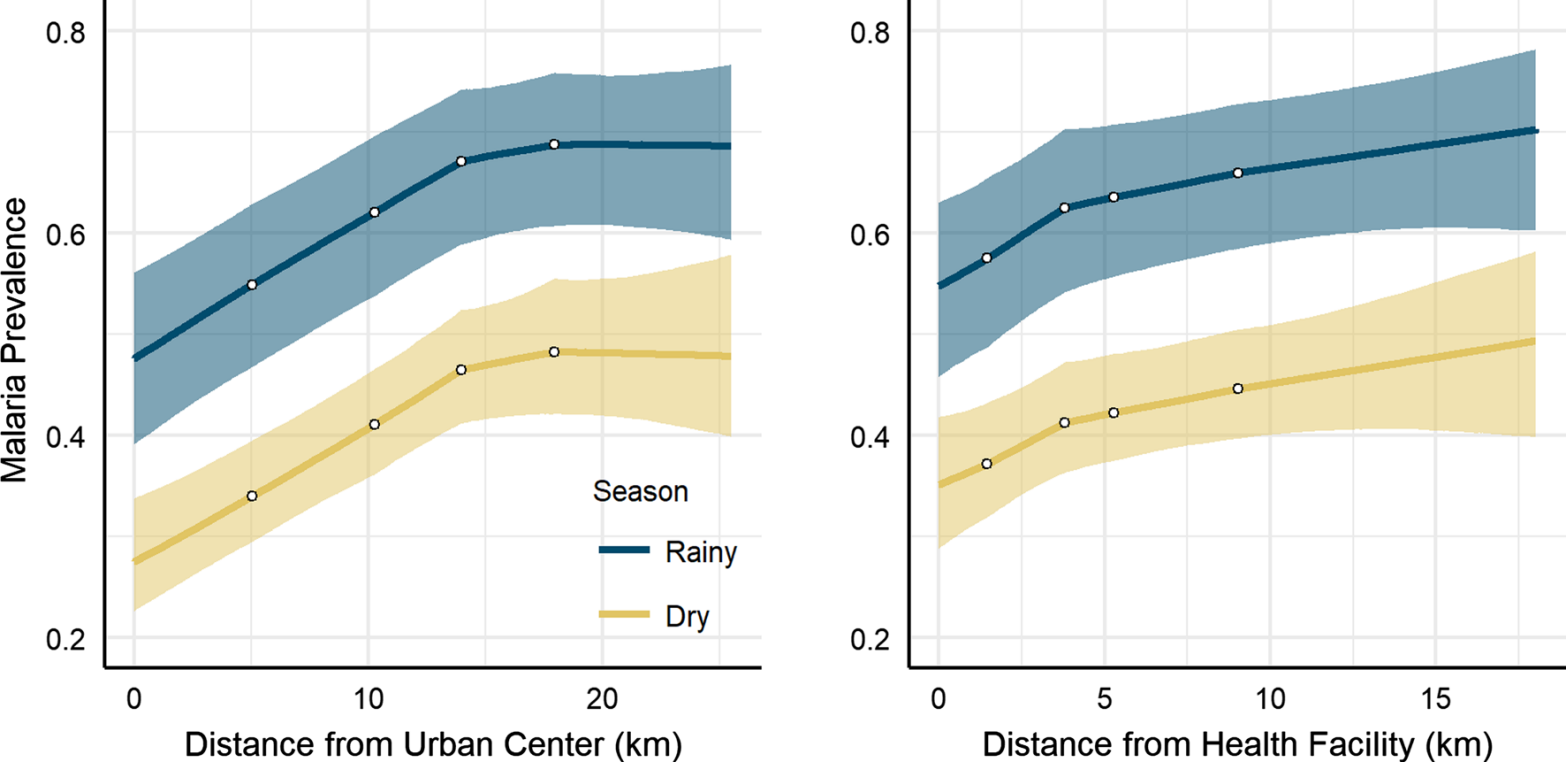
Implied patterns in malaria prevalence and distance to urban center (left) and distance to health facility (right) based on Bayesian probit regression model containing linear splines and seasonal interactions. Results for the rainy and dry seasons are shown in blue and yellow, respectively. The open circles depict where slopes are allowed to change (i.e., knot locations), selected at 20% quantiles of the observed data

The final model containing linear spline covariates revealed interesting nonlinear associations between malaria prevalence and distance to nearest urban centre, and distance to nearest health facility (Fig. 4). Distance to nearest urban centre was positively associated with malaria infection in a roughly linear pattern until about 12–14 kilometres (km), after which malaria risk began to plateau. Similarly, the implied malaria risk was greater for communities that were further away from the nearest health facilities, however there was a less steep relationship after approximately 2–4 km. These nonlinear patterns in malaria risk and proximity to urban centres and health facilities were consistent in the rainy and dry seasons.

### Out-of-sample predictions

**Fig. 5:**
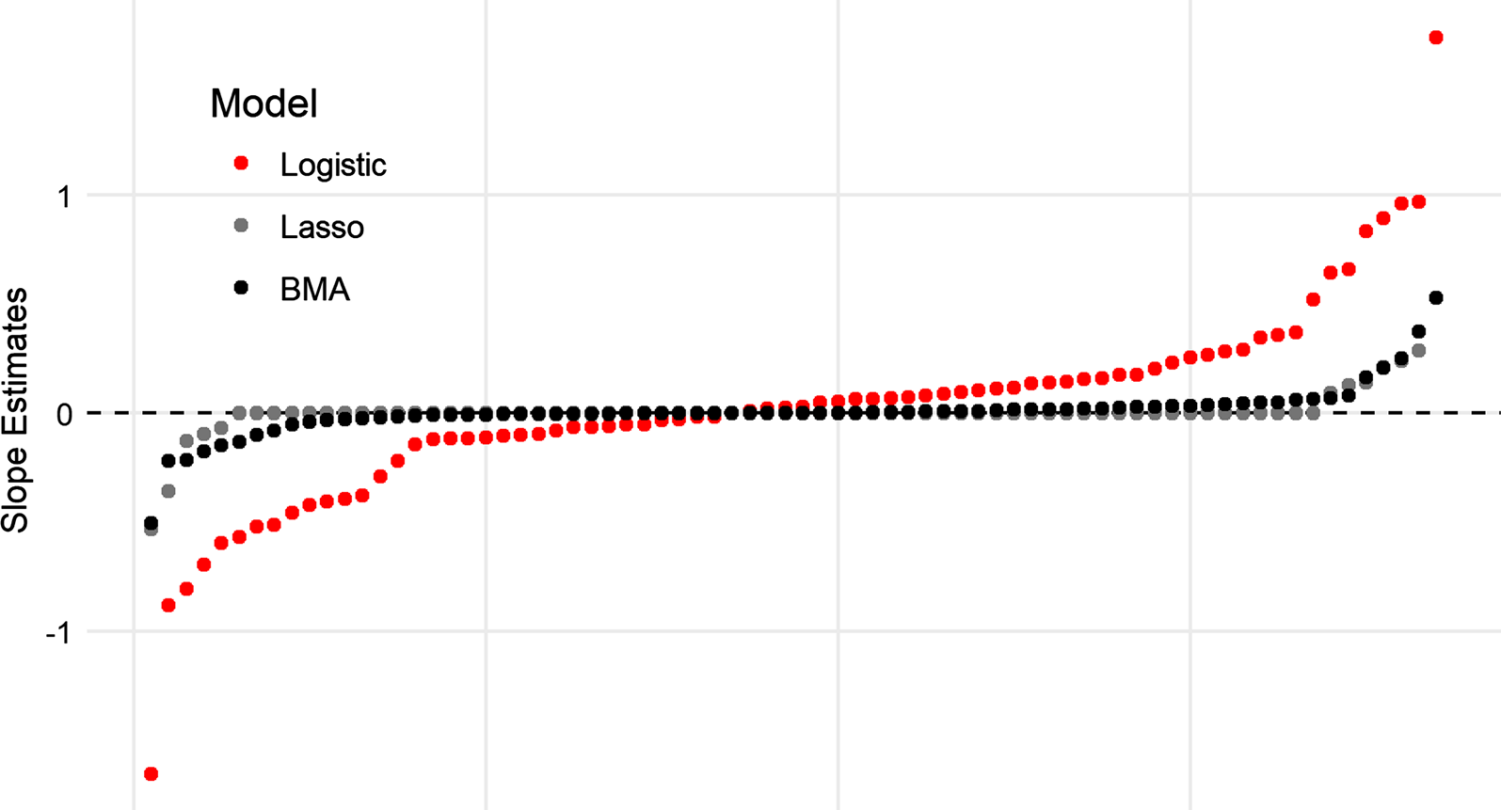
Regression parameter estimates using BMA (black), logistic regression (red), and Lasso regression (gray) models containing interactions terms and spline covariates (73 independent variables). Parameter estimates were ordered according to the logistic regression results to better illustrate the shrinkage of coefficients associated with the BMA and Lasso algorithms

Based on the sum of the log-likelihood, BMA and Lasso regression both outperformed standard logistic regression for all predictions (Table 3). Both approaches improved the out-of-sample predications compared to standard logistic regression by shrinking the regression coefficient estimates towards zero (Fig. 5). A notable difference between these approaches is that BMA allows for near-zero parameter estimates, whereas Lasso will force marginal factors to zero. For the base set of covariates, BMA and Lasso had similar likelihood values, however BMA had higher likelihood values for all predictions based on the extended set of covariates, which included seasonal interactions and linear splines. In particular, note that the out-of-sample predictive skill of BMA increased slightly for the extended model relative to the base model whereas the predictive skill of the logistic regression model and the Lasso often (or always) decreased when comparing these models. These results suggest that BMA is noticeably more resistant to overfitting than Lasso or logistic regression as the number of parameters is substantially increased.

**Table 3:** Predictive comparisons of models based on the sum of the log-likelihood

Training	Testing	Sum of log-likelihood		
		Logistic	Lasso	BMA
Base model (p = 29)^a^				
Rainy 2010	Rainy 2011	− 1072.27	− 1049.24	− 1028.24^b^
Rainy 2011	Rainy 2012	− 1055.39	− 1037.49	− 1032.57^b^
Rainy 2010	Rainy 2012	− 1153.62	− 1110.05	− 1057.07^b^
Dry 2011	Dry 2012	− 969.88	− 919.45^b^	− 921.54
Dry 2012	Dry 2013	− 915.95	− 897.03^b^	− 903.60
Dry 2011	Dry 2013	− 967.83	− 920.28	− 915.81^b^
	Average	− 1022.49	− 988.92	− 976.47
Model with interactions and splines (p = 73)^a^				
Rainy 2010	Rainy 2011	− 1079.63	− 1042.02	− 1027.85^b^
Rainy 2011	Rainy 2012	− 1066.56	− 1035.44	− 1030.75^b^
Rainy 2010	Rainy 2012	− 1156.76	− 1092.52	− 1050.55^b^
Dry 2011	Dry 2012	− 1065.27	− 1029.66	− 921.05^b^
Dry 2012	Dry 2013	− 922.40	− 902.79	− 902.32^b^
Dry 2011	Dry 2013	− 1079.34	− 1059.24	− 917.82^b^
	Average	− 1061.66	− 1026.95	− 975.06

*BMA* Bayesian model average

^a^p refers to the number of covariates in the model

^b^Indicates the model with the best fit

## Discussion

### Methodological findings

These findings lend strong support for the usefulness of Bayesian model averaging (BMA) as a statistical tool for detecting complex patterns in malaria risk factors. In order to promote reproducibility of these methods and findings, the code used to run this analysis has been provided in Additional file 1 and have placed the data and R scripts in a public repository (see “Availability of data and materials” section), and note that packages for similar model selection and averaging approaches using OpenBUGS and R are available [53, 54]. Moreover, BMA in this context demonstrated similar advantages over standard variable selection procedures found in simulation studies [30–32], and studies in other ecological contexts [32–37, 55], including epidemiological risk analysis [38, 39]. Unlike standard logistic and Lasso regression, increasing model complexity by including several additional covariates did not reduce the out-of-sample predictive performance when using BMA. Importantly, constructing confidence intervals for Lasso regression coefficients continues to be an ongoing area of research [56–58], whereas characterizing uncertainty via credible intervals in the Bayesian framework is straightforward. Credible intervals are also often a better approach for comparing the strength of associations when compared to other traditional metrics, such as p-values [59]. Reversible jump MCMC tends to be computational efficient and effective, and by integrating out the regression coefficients this Gibbs sampler avoided issues associated with poor mixing of chains that often plague these other variable selection approaches [32, 53]. Machine learning techniques, such as artificial neural networks and support vector machines, can also detect nonlinear and other relationships and often have better predictive performance than standard logistic regression, but it is typically difficult to make direct inferences about the role of individual covariates using these techniques [60]. BMA may be useful for specific applications to modelling malaria risk factors where both interpretability and predictive ability are important (such as designing locally targeted interventions).

The trade-off between interpretability and predictive skill, spatial and temporal scope, data accessibility, and computational limitations are important factors to consider when choosing a variable selection procedure. For example, Weiss et al. [23] describes an exhaustive analysis of variable selection for identifying environmental factors associated with *Plasmodium falciparum* prevalence across sub-Saharan Africa, containing over 50 million covariates. They then used a series of selection phases based on Akaike information criteria (AIC) to reduce the number of covariates. This procedure was able to distill a parameter space that would be computational impossible to explore using BMA, however it lacks interpretability and does not account for uncertainty in the selection process. These tradeoffs may not be significant for prediction applications, but are critical for the analysis in this study to appropriately generate inference on the significance of different predictor variables.

### Inferences on malaria risk factors and control strategies

Another area for further analysis is utilizing the model interpretability characteristics of this framework for informing management applications. The BMA-based analysis described well-established patterns in malaria aetiology across sub-Saharan Africa, including the strong seasonal patterns in malaria transmission [40, 41] and age-related prevalence patterns [61]. Protective factors identified in our models, including access to health insurance and mother’s education, have also been described as important factors in similar settings [62, 63]. In addition to validating these data and the methodology, these findings may provide insight for guiding local intervention and control strategies. For instance, the protective effect of personal health insurance coverage, which was detectable in one of Ghana’s more remote corners, underscores the value of Ghana’s pioneering effort to institute and scale up a national health insurance scheme since 2006 [64].

The capacity to increase model complexity without sacrificing predictive performance is an important modelling characteristic, particularly when inference is used to inform management strategies [65]. The inclusion of seasonal interaction terms revealed seasonal differences in age- and ethnicity-related risk that may be useful for designing seasonal chemoprophylaxis interventions, which can be extremely effective method for reducing cost and maximizing impact depending on the local malaria dynamics [66–69]. The linear spline covariates allowed the model to describe the nonlinear protective buffer provided by the modest urban centers. The link between urbanicity and malaria transmission has been extensively discussed in the literature [70–73], but understanding the relative impact modest urban centers can have on health outcomes in rural regions can be challenging [74], particularly at small spatial scales. The revealed nonlinear relationship between malaria risk and distance to urban centers suggested that the risk associated with living far from the urban center eventually reaches a plateau around 12 km in Bunkpurugu-Yunyoo. This is an interesting finding considering that the increased housing density, reduced non-polluted water resources, and other urban characteristics resolved about 2–3 km from the centers of the towns, based on field observation and satellite imagery. In addition, IRS coverage was universal across the district and ITN use was the same or higher at the more remote locations. This implies that ecological and entomological factors are less likely to be driving this phenomenon, suggesting that socio-economic factors may be important.

Furthermore, this framework may be useful for projecting the impact of future management efforts. For example, the association between malaria risk and distance to nearest health facility became less pronounced after about 2–4 km. Comparable rate stabilization patterns at similar distances have been described in health facilities in rural regions of Kenya [75]. Distance to nearest health facility is known to be an important factor in treatment-seeking behaviour and health outcomes [76–78]. Access to healthcare is a guiding management principle in Ghana, as demonstrated by the expansion of access to health insurance and revitalization of the Community-Based Health Planning and Services (CHPS) programme. Future work with these data will build upon these findings to describe the impact of CHPS facilities on early childhood malaria in Bunkpurungu-Yunyoo, as well as project the potential impact of new CHPS facilities and optimize their locations.

Bayesian model selection approaches, like BMA, are likely to find its greatest value in forecasting applications, in which model interpretability, predictive performance, and uncertainty characterization are equally valued. Bayesian frameworks often require a deeper understanding in statistical theory and programming, can be computationally intensive, and may lack accessible tools/software, but offer many advantages for modelling epidemiological data, including high flexibility and intuitive expressions of inference and uncertainty [79]. Based on background literature review, this appears to be the first instance of using BMA for variable selection to model malaria risk factors. This methodology offers a flexible framework with many advantages over other methods for modelling disease risk factors.

### Limitations

From a methodological perspective, the outcomes from this study provide promising support for BMA as a useful statistical tool for modelling highly dimensional data on malaria risk factors, however there are notable limitations. The analysis uses a single data set, and therefore further efforts are needed to corroborate these findings. It may be that at certain dimensionalities BMA less effective than the other methods tested in this article, and therefore this analyses should be applied to other data sets, particularly at different spatio-temporal scales. Other comparison criteria (such as area under the receiver operating curve) or other tests (such as cross-validation) could also be used to compare predictive performance. From an epidemiological perspective, while this study incorporates many potential risks for malaria there are additional variables that are not included. Most notably these data do not include vector-related variables. The data are also limited by the periodicity of sampling time (seasonal), rather than a continuous sampling approach.

## Conclusion

The BMA approach for variable selection produced easily interpretable models, which incorporate selection uncertainty and outperformed standard logistic and Lasso regressions in out-of-sample predictions. The risk factor models for malaria prevalence in young children from a holoendemic district in northern Ghana experiencing residual transmission revealed complex patterns of disease drivers, including nonlinear relationships between malaria status and distance from the nearest urban centre and health facility, as well as seasonal differences in risk associated with age and ethnicity. Models quickly become increasingly more complex with additional explanatory variables (and their associated parameters) to increase flexibility, underscoring the need for reliable methods for model selection. Bayesian approaches for variable selection, such as BMA, for identifying and describing risk factor have potential for expanding the understanding of local drivers of disease, leading to more efficient targeting and prioritization of existing interventions, and informing new interventions, for malaria and other vector-borne diseases.

## Additional file

**Additional file 1.** Contains descriptive statistics on covariates, code for running the Gibbs sampler, and additional model outputs.

## Abbreviations

BMA: Bayesian model averaging
Comm.: community
Edu.: education
GIS: geographic information systems
IRS: indoor-residual spraying of insecticides
ITN: insecticide-treated bed netting
Km: kilometres
Lasso: least absolute shrinkage and selection operator
LLIN: long-lasting insecticide-treated bed net
LST: land surface temperature
NDVI: normalized difference vegetation index
WQ: wealth quintile
