# Supplementary material for: Detecting local risk factors for residual malaria in northern Ghana using Bayesian model averaging
Source: Malar J. 2018 Sep 29;17:343. doi: 10.1186/s12936-018-2491-2 (PMC6162921; doi:10.1186/s12936-018-2491-2)
Supplement: Supplementary file 1 — Additional file 1. Contains descriptive statistics on covariates, code for running the Gibbs sampler, and additional model outputs. [file 12936_2018_2491_MOESM1_ESM.pdf]

Additional file 1

Contents

1 Descriptive Analysis **1**

2 Gibbs Sampler Code **4**
2.1 Gibbs Functions 4
2.2 Gibbs Sampler 7

3 Model Outputs **8**
3.1 Regression coefficient tables 8
3.2 Interaction model coefficent plot 11

1 Descriptive Analysis

Malaria status was assessed in young children (ages 6 to 59 months old) in six biannual seasonal surveys. Average community prevalence was significantly higher in the rainy season (0.516) than in the dry season (0.298). Seasonal prevalence rates slightly decreased on average over the course of the study, however this was not a significant shift (Fig. 1). Based on this finding, seasonal risk was modelled as a single binary variable (i.e. 1 if observation was collected during the rainy season, and 0 otherwise). This binary covariate was also used to construct seasonal interaction terms.

In order to address issues that could arise from collinearity, a correlation matrix between all potential covariates (i.e. risk factors) was constructed (Fig. 2). For instances where two covariates had a high degree of correlation ($R^2 > 0.49$), a single representative covariate was selected based on the relevance of each covariate to malaria epidemiology and intervention strategies. The covariates dropped from all models were farming households, indoor residual spraying (IRS) in past 7 months, average daytime land surface temperature, normalized difference vegetation index (NDVI), cumulative rainfall, and historical precipitation trends.

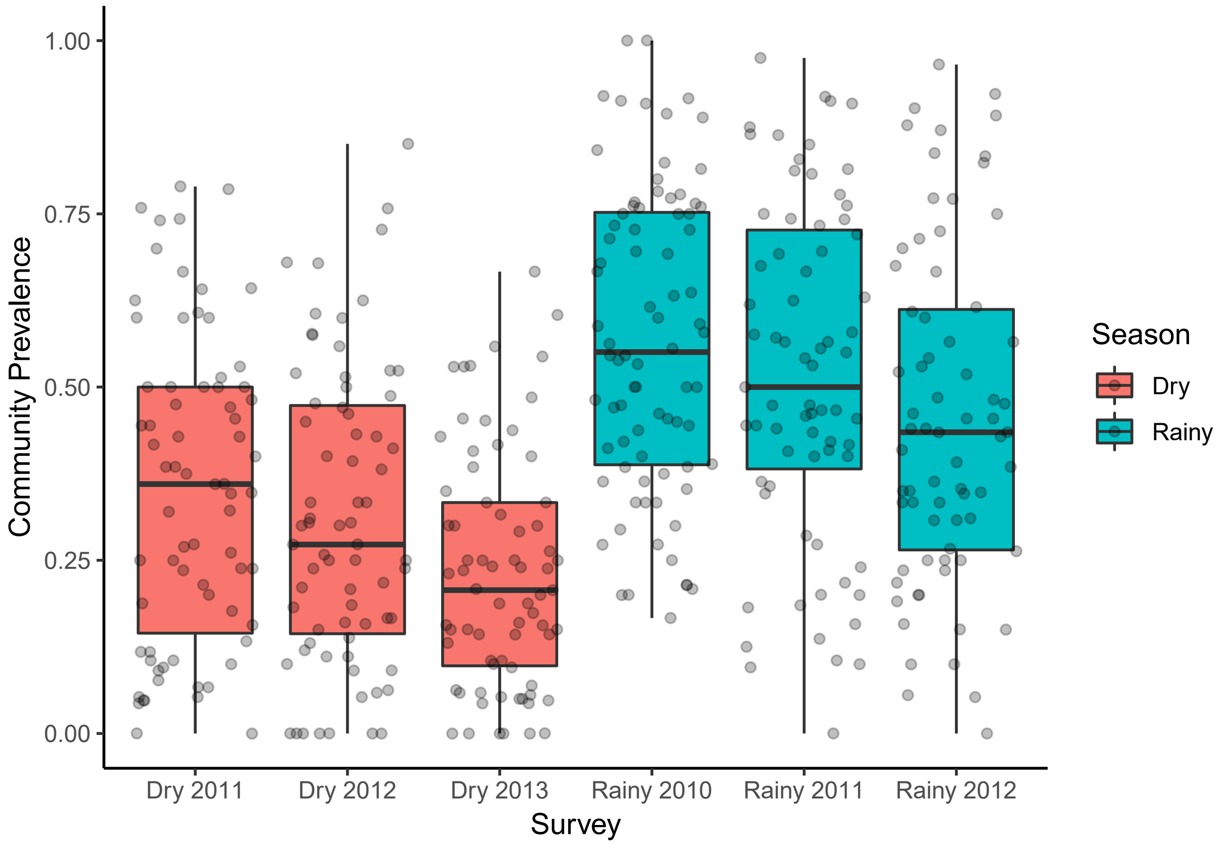

Figure S 1 : Mean community malaria prevalence by survey. Black circles represent individual communities, boxes display distribution of community prevalences within a survey.

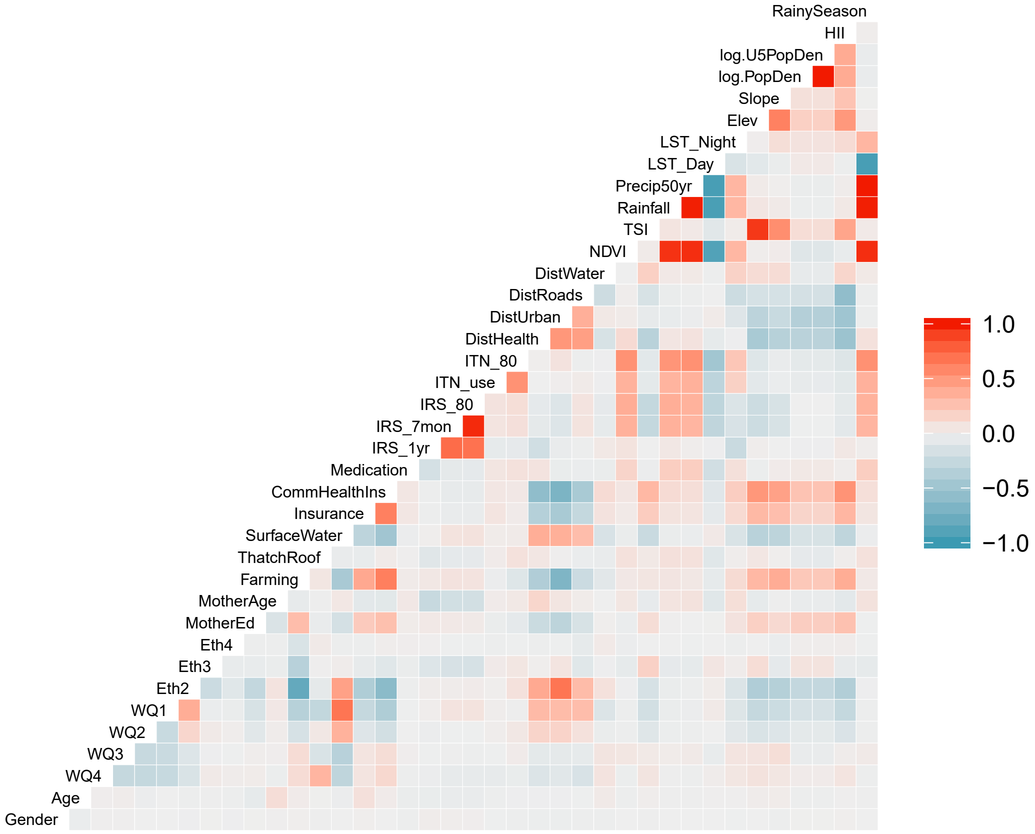

Figure S2: Covariate correlation matrix.

2 Gibbs Sampler Code

2.1 Gibbs Functions

The following code contains the functions used in the Gibbs sampler:

```
tnorm <- function(n,lo,hi,mu,sig){
  #generates truncated normal variates based on cumulative normal distribution
  #normal truncated lo and hi

  if(length(lo) == 1 & length(mu) > 1)lo <- rep(lo,length(mu))
  if(length(hi) == 1 & length(mu) > 1)hi <- rep(hi,length(mu))

  q1 <- pnorm(lo,mu,sig) #cumulative distribution
  q2 <- pnorm(hi,mu,sig) #cumulative distribution

  z <- runif(n,q1,q2)
  z <- qnorm(z,mu,sig)
  z[z == -Inf]  <- lo[z == -Inf]
  z[z == Inf]   <- hi[z == Inf]
  z
}
#-------------------------------
rmvnorm=function (n, mean = rep(0, nrow(sigma)), sigma = diag(length(mean)),
                  method = c("eigen", "svd", "chol"))
{
  if (!isSymmetric(sigma, tol = sqrt(.Machine$double.eps),
                   check.attributes = FALSE)) {
    stop("sigma must be a symmetric matrix")
  }
  if (length(mean) != nrow(sigma)) {
    stop("mean and sigma have non-conforming size")
  }
  sigma1 <- sigma
  dimnames(sigma1) <- NULL
  if (!isTRUE(all.equal(sigma1, t(sigma1)))) {
    warning("sigma is numerically not symmetric")
  }
  method <- match.arg(method)
  if (method == "eigen") {
    ev <- eigen(sigma, symmetric = TRUE)
    if (!all(ev$values >= -sqrt(.Machine$double.eps) * abs(ev$values[1]))) {
      warning("sigma is numerically not positive definite")
    }
    retval <- ev$vectors %*% diag(sqrt(ev$values), length(ev$values)) %*%
      t(ev$vectors)
  }
  else if (method == "svd") {
    sigsvd <- svd(sigma)
    if (!all(sigsvd$d >= -sqrt(.Machine$double.eps) * abs(sigsvd$d[1]))) {
      warning("sigma is numerically not positive definite")
    }
    retval <- t(sigsvd$v %*% (t(sigsvd$u) * sqrt(sigsvd$d)))
  }
```

```r
  else if (method == "chol") {
    retval <- chol(sigma, pivot = TRUE)
    o <- order(attr(retval, "pivot"))
    retval <- retval[, o]
  }
  retval <- matrix(rnorm(n * ncol(sigma)), nrow = n) %*% retval
  retval <- sweep(retval, 2, mean, "+")
  colnames(retval) <- names(mean)
  retval
}
#------------------------------
update.betas=function(param){
  p=ncol(param$cov)
  Sigma.inv=diag(x=1,p)
  Sigma.inv[1,1]=1/1000
  prec=t(param$cov)%*%param$cov+(1/param$sigma2)*Sigma.inv
  var1=solve(prec)
  pmedia=t(param$cov)%*%param$z
  rmvnorm(1,var1%*%pmedia,var1)
}
#------------------------------
update.sigma2=function(param){
  p=ncol(param$cov)
  a1=(p-1)/2

  Sigma.inv=diag(x=1,p)
  Sigma.inv[1,1]=1/1000
  b1=(t(param$betas)%*%Sigma.inv%*%param$betas)/2
  1/rgamma(1,a1,b1)
}
#------------------------------
update.z=function(param){
  cond=dat$y>0
  media=param$cov%*%param$beta
  res=rep(NA,n)
  res[cond]=tnorm(sum(cond),lo=0,hi=Inf,mu=media[cond],sig=1)
  res[!cond]=tnorm(sum(!cond),lo=-Inf,hi=0,mu=media[!cond],sig=1)
  res
}
#------------------------------
log.marg.likel=function(cov,z,sig2){
  w=z
  p=ncol(cov)
  Sigma.inv=diag(x=1,p)
  Sigma.inv[1,1]=1/1000
  prec=t(cov)%*%cov+(1/sig2)*Sigma.inv
  var1=solve(prec)
  mu=var1%*%t(cov)%*%w
  -(p/2)*log(sig2)-(1/2)*(-t(mu)%*%prec%*%mu)+(1/2)*determinant(var1)$modulus[1]
}
#------------------------------
samp.move=function(paramz){
  indin.old=paramz$indin
```

```
  p=length(indin.old)
  z=runif(1)
  p0=1
  if (p == 1) {
    indin.new=birth(paramz$indin,paramz$indout)
    # death prob 2 -> 1 is (1/3) and birth prob 1 -> 2 is 1.
    p0=1/3
  }
  if (p == maxp) {
    if (z < 1/2) {
      indin.new=death(paramz$indin)
      # birth prob T-1 -> T is (1/3) and death prob T -> T-1 is 1/2
      p0=2/3
    }
    if (z >= 1/2) indin.new=swap(paramz$indin,paramz$indout)
  }
  if (1 < p & p < maxp) {
    if (z < 1/3) {
      indin.new=birth(paramz$indin,paramz$indout)
      # death prob from T -> T-1 is (1/2) and birth prob from T-1 -> T is (1/3)
      if (p==maxp-1) p0=3/2
    }
    if (1/3 < z & z < 2/3) {
      indin.new=death(paramz$indin)
      # birth prob from 1 -> 2 is 1 and death prob from 2 -> 1 is 1/3
      if (p==2) p0=3
    }
    if (2/3 < z) indin.new=swap(paramz$indin,paramz$indout)
  }
  pold=log.marg.likel(xmat.orig[,indin.old],paramz$z,paramz$sigma2)
  pnew=log.marg.likel(xmat.orig[,indin.new],paramz$z,paramz$sigma2)+log(p0)
  prob=exp(pnew-pold)
  z=runif(1)

  seq1=1:maxp
  k=which(!seq1%in%indin.new)
  indout.new=seq1[k]
  if (z<prob) return(list(xmat=xmat.orig[,indin.new],indin=indin.new,
                         indout=indout.new))
  return(list(xmat=xmat.orig[,indin.old],indin=indin.old,indout=paramz$indout))
}
#------------------------------
death=function(indinz){
  if (length(indinz)==2) return(indinz[2]) # cannot delete intercept
  k=sample(2:length(indinz),size=1)
  indinz[-k]
}
#------------------------------
swap=function(indinz,indoutz){
  if (length(indinz)==2) k=indinz[2] # cannot swap intercept
  if (length(indinz)!=2) k=sample(2:length(indinz),size=1)
  tmp=indinz[-k]
  include=sample(indoutz,size=1)
```

```
  sort(c(tmp,include))
}
#-------------------------------
birth=function(indinz,indoutz){
  k=sample(indoutz,size=1)
  sort(c(indinz,k))
}
#-------------------------------
```

These functions were used for the base model. The models containing seasonal interaction terms contained a single alteration which did not allow the seasonal intercept to be removed from the model.

2.2 Gibbs Sampler

The following code is used to initialize and run the Gibbs sampler:

```
rm(list=ls(all=T))
set.seed(1)
source('scripts/functions.R')
dat=read.csv('datafiles/GibbsInput.csv',as.is=T)

nvillage=length(unique(dat$village))
npeople.village=table(dat$village)
n=nrow(dat)

ind=grep('cov',colnames(dat))
xmat.orig=cov=data.matrix(cbind(1,dat[,ind]))
maxp=ncol(cov)

# initial values
betas=c(0.2,rep(0,maxp-1))
sigma2=1
cond=dat$y>0
z=ifelse(cond,runif(n),runif(n,min=-1,max=0))
indin=1:10
indout=11:maxp
param=list(z=z,sigma2=sigma2,betas=matrix(betas[indin],length(indin),1),
           indin=indin,indout=indout,cov=cov[,indin])

ngibbs=10000
vec.betas=matrix(NA,ngibbs,maxp)
vec.outros=matrix(NA,ngibbs,1)

# run gibbs
for (i in 1:ngibbs){
  print(c(i,param$indin))
  if (!1%in%param$indin) break;
  tmp=samp.move(param)
  param$cov=tmp$xmat
  param$indin=tmp$indin
  param$indout=tmp$indout

  param$betas=t(update.betas(param))
```

```
  param$sigma2=update.sigma2(param)
  param$z=update.z(param)

  tmp=rep(0,maxp)
  tmp[param$indin]=param$betas
  vec.betas[i,]=tmp
  vec.outros[i,]=param$sigma2

}
```

3 Model Outputs

3.1 Regression coefficient tables

3.1.1 Base model

Table S1: Base model regression coefficents

Covariate	Slope Est.	Lower 95% C.I.	Upper 95% C.I.
Intercept	-0.269	-0.156	-0.378
Gender	0.001	0.032	-0.025
Age	0.296	0.324	0.268
WQ4	0.016	0.106	-0.025
WQ3	0.014	0.109	-0.025
WQ2	0.092	0.229	0.000
WQ1	0.165	0.334	0.000
Eth2	0.233	0.323	0.137
Eth3	0.103	0.228	0.000
Eth4	-0.013	0.146	-0.208
MotherEd	-0.220	-0.141	-0.300
MotherAge	0.033	0.066	0.000
ThatchRoof	-0.012	0.014	-0.082
SurfaceWater	-0.056	0.036	-0.194
Insurance	-0.463	-0.391	-0.530
CommHealthIns	-0.007	0.014	-0.058
Medication	0.012	0.081	-0.010
IRS_1yr	-0.154	-0.050	-0.254
IRS_80	0.083	0.192	0.000
ITN_use	-0.031	0.001	-0.121
ITN_80	0.011	0.091	-0.020
DistHealth	0.094	0.131	0.056
DistUrban	0.183	0.229	0.137
DistRoads	0.014	0.060	0.000
DistWater	-0.013	0.000	-0.052
LST_Night	-0.005	0.005	-0.039
Elev	-0.187	-0.142	-0.231
Slope	0.082	0.120	0.045
log.PopDen	-0.001	0.009	-0.024
RainySeason	0.647	0.728	0.565

[1] Bolded covariates were considered statistically significant.

[2] Abbreviations: WQ - wealth quintile, Eth2 - Konkomba, Eth3 - Mamprusi, Eth4 - Other, Insurance - personal health insurance, IRS_1yr - indoor residual spraying in past year, IRS_80 - indoor residual spraying community coverage over 80%, ITN_use - insecticide treated netting, ITN_80 - insecticide treated netting community coverage over 80%, LST_Night - land surface temperature at night, Elev - elevation, log.PopDen - log-transformed population density.

3.1.2 Seasonal interactions model

Table S2: Seasonal interaction model regression coefficients

	Dry Season			Rainy Season		
Covariate	Slope Est.	Lower 95% C.I.	Upper 95% C.I.	Slope Est.	Lower 95% C.I.	Upper 95% C.I.
Intercept	-0.220	-0.076	-0.360	-0.025	0.324	-0.362
Gender	-0.003	0.029	-0.055	0.008	0.115	-0.094
Age	0.371	0.411	0.331	0.594	0.672	0.515
WQ4	0.027	0.143	-0.026	0.040	0.260	-0.109
WQ3	-0.002	0.084	-0.100	0.047	0.258	-0.130
WQ2	0.068	0.212	-0.006	0.169	0.462	-0.012
WQ1	0.106	0.292	-0.002	0.287	0.678	-0.002
Eth2	0.143	0.277	0.000	0.435	0.693	0.170
Eth3	0.059	0.201	-0.028	0.148	0.447	-0.046
Eth4	-0.012	0.138	-0.197	-0.027	0.335	-0.433
MotherEd	-0.217	-0.126	-0.314	-0.425	-0.243	-0.612
MotherAge	0.071	0.125	0.000	0.087	0.181	-0.022
ThatchRoof	-0.024	0.019	-0.109	-0.058	0.047	-0.229
SurfaceWater	-0.035	0.053	-0.176	-0.087	0.116	-0.379
Insurance	-0.494	-0.398	-0.606	-0.921	-0.727	-1.121
CommHealthIns	-0.059	0.000	-0.155	-0.040	0.091	-0.202
Medication	0.073	0.207	0.000	0.058	0.268	-0.100
IRS_1yr	-0.153	0.000	-0.273	-0.238	0.118	-0.525
IRS_80	0.072	0.204	-0.008	0.116	0.418	-0.166
ITN_use	-0.039	0.005	-0.135	-0.047	0.123	-0.252
ITN_80	0.004	0.088	-0.073	0.114	0.323	-0.073
DistHealth	0.081	0.133	0.000	0.192	0.299	0.065
DistUrban	0.180	0.233	0.125	0.360	0.469	0.249
DistRoads	0.005	0.046	-0.004	0.020	0.113	-0.013
DistWater	-0.024	0.000	-0.066	-0.049	0.000	-0.132
LST_Night	-0.022	0.000	-0.076	0.028	0.128	-0.098
Elev	-0.166	-0.101	-0.223	-0.341	-0.222	-0.454
Slope	0.064	0.115	0.000	0.159	0.268	0.043
log.PopDen	-0.002	0.005	-0.030	-0.004	0.029	-0.062

[1] Bolded covariates were considered statistically significant in the dry and rainy seasons.
[2] Italicized covariates were considered statistically significant only in the rainy season.
[3] The regression intercept was significant only in the dry season.
[4] Abbreviations: see Table 1

3.1.3 Linear spline model

Table S 3 : Linear spline model regression coefficents

	Dry Season			Rainy Season		
Covariate	Slope Est.	Lower 95% C.I.	Upper 95% C.I.	Slope Est.	Lower 95% C.I.	Upper 95% C.I.
Intercept	-0.219	-0.082	-0.341	0.311	0.655	-0.034
Gender	0.000	0.018	-0.013	0.004	0.082	-0.020
Age	0.371	0.413	0.331	0.223	0.320	0.127
WQ4	0.001	0.032	-0.006	-0.006	0.062	-0.108
WQ3	-0.003	0.009	-0.061	0.013	0.151	-0.065
WQ2	0.018	0.147	0.000	0.033	0.291	-0.026
WQ1	0.060	0.223	0.000	0.106	0.453	-0.003
Eth2	0.207	0.335	0.000	0.287	0.653	0.000
Eth3	0.030	0.185	0.000	0.049	0.366	-0.051
Eth4	-0.007	0.106	-0.166	-0.014	0.254	-0.370
MotherEd	-0.216	-0.130	-0.309	-0.207	0.001	-0.371
MotherAge	0.069	0.127	0.000	0.017	0.127	-0.128
ThatchRoof	-0.017	0.000	-0.100	-0.028	0.000	-0.215
SurfaceWater	-0.004	0.033	-0.093	-0.003	0.123	-0.188
Insurance	-0.503	-0.408	-0.614	-0.439	-0.178	-0.614
CommHealthIns	-0.023	0.000	-0.138	0.006	0.178	-0.138
Medication	0.024	0.185	0.000	-0.006	0.185	-0.235
IRS_1yr	-0.130	0.000	-0.259	-0.086	0.260	-0.275
IRS_80	0.040	0.189	0.000	0.040	0.327	-0.170
ITN_use	-0.042	0.000	-0.144	-0.027	0.159	-0.186
ITN_80	0.000	0.058	-0.062	0.046	0.261	-0.062
DistHealth	0.163	0.394	0.000	0.199	0.681	-0.011
DistUrban	0.249	0.397	0.126	0.249	0.490	0.031
DistRoads	0.007	0.052	0.000	0.009	0.096	0.000
DistWater	-0.003	0.000	-0.038	-0.004	0.006	-0.059
LST_Night	-0.008	0.000	-0.068	0.012	0.134	-0.068
Elev	-0.175	-0.121	-0.227	-0.176	-0.098	-0.273
Slope	0.032	0.090	0.000	0.044	0.184	0.000
log.PopDen	0.000	0.010	-0.017	0.000	0.025	-0.018
Spline Covariates						
DistHealth.s1	0.029	0.320	-0.256	0.035	0.523	-0.436
DistHealth.s2	-0.100	0.027	-0.318	-0.129	0.078	-0.601
DistHealth.s3	-0.003	0.120	-0.127	-0.003	0.216	-0.228
DistHealth.s4	-0.005	0.000	-0.070	-0.007	0.023	-0.124
DistUrban.s1	-0.006	0.129	-0.175	-0.009	0.212	-0.294
DistUrban.s2	0.007	0.158	-0.100	0.007	0.228	-0.171
DistUrban.s3	-0.078	0.000	-0.204	-0.076	0.081	-0.261
DistUrban.s4	-0.022	0.002	-0.103	-0.020	0.039	-0.103

[1] Bolded covariates were considered statistically significant in the dry and rainy seasons.
[2] Italicized covariates were considered statistically significant only in the dry season.
[3] Abbreviations: see Table 1, .s1 - first spline knot

3.2 Interaction model coefficent plot

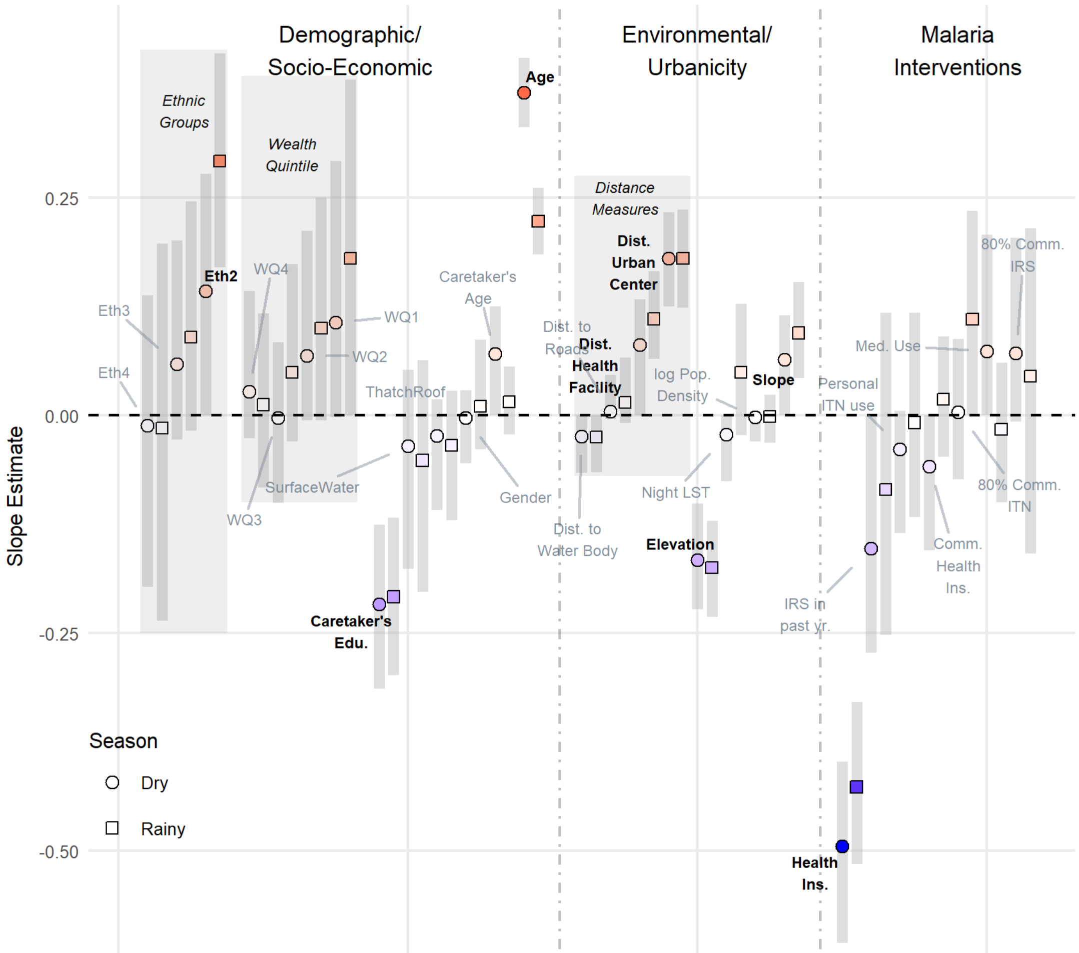

Figure S3: Mean slope estimates and 95% credible intervals (horizontal grey bars) from probit regression parameters. Seasonal risk coefficent are labelled by shape. Variables whose 95% credible intervals do not include zero are considered significant (labelled in bold). Risk and protective factors are shown in red and blue, respectively.
